## [Peer Review History · The Journal of Physiology]

Impact of cancer cachexia on respiratory muscle function and the therapeutic potential of exercise

Ken D O'Halloran, John J Mackrill, and Ben Murphy

DOI: 10.1113/JP283569

Corresponding author(s): Ken O'Halloran (k.ohalloran@ucc.ie)

The following individual(s) involved in review of this submission have agreed to reveal their identity: Russell T. Hepple (Referee #1)

Review Timeline:

Submission Date:	18-Jul-2022
Editorial Decision:	16-Aug-2022
Revision Received:	29-Aug-2022
Accepted:	09-Sep-2022

Senior Editor: Laura Bennet

Reviewing Editor: Scott Powers

Transaction Report:

Dear Professor O'Halloran,

Re: JP-TR-2022-283569 "The impact of cancer cachexia on respiratory muscle function and the therapeutic potential of exercise" by Ken D O'Halloran, John J Mackrill, and Ben Murphy

Thank you for submitting your Topical Review to The Journal of Physiology. It has been assessed by a Reviewing Editor and by 2 expert referees and I am pleased to tell you that it is considered to be acceptable for publication following satisfactory revision.

The reports are copied at the end of this email. Please address all of the points and incorporate all requested revisions, or explain in your Response to Referees why a change has not been made.

NEW POLICY: In order to improve the transparency of its peer review process The Journal of Physiology publishes online as supporting information the peer review history of all articles accepted for publication. Readers will have access to decision letters, including all Editors' comments and referee reports, for each version of the manuscript and any author responses to peer review comments. Referees can decide whether or not they wish to be named on the peer review history document.

I hope you will find the comments helpful and have no difficulty in revising your manuscript within 4 weeks.

Your revised manuscript should be submitted online using the links in Author Tasks: Link Not Available. This link is to the Corresponding Author's own account, if this will cause any problems when submitting the revised version please contact us.

You should upload:

- A Word file of the complete text (including any Tables);
- An Abstract Figure, (with accompanying Legend in the article file)
- Each figure as a separate, high quality, file;
- A full Response to Referees;
- A copy of the manuscript with the changes highlighted.
- Author profile. A short biography (no more than 100 words for one author or 150 words in total for two authors) and a portrait photograph of the two leading authors on the paper. These should be uploaded, clearly labelled, with the manuscript submission. Any standard image format for the photograph is acceptable, but the resolution should be at least 300 dpi and preferably more.

- A 'Cover Art' file for consideration as the Issue's cover image;
- Appropriate Supporting Information (Video, audio or data set https://jp.msubmit.net/cgi-bin/main.plex?form_type=display_requirements#supp).

To create your 'Response to Referees' copy all the reports, including any comments from the Senior and Reviewing Editors into a Word, or similar, file and respond to each point in colour or CAPITALS. Upload this when you submit your revision.

I look forward to receiving your revised submission.

Yours sincerely,

Professor Laura Bennet
Senior Editor
The Journal of Physiology
<https://jp.msubmit.net>
<http://jp.physoc.org>
The Physiological Society
Hodgkin Huxley House
30 Farringdon Lane
London, EC1R 3AW
UK
<http://www.physoc.org>
<http://journals.physoc.org>

EDITOR COMMENTS

Reviewing Editor:

Thank you for submitting this topical review to the Journal of Physiology. Your report has been reviewed by two experts in the field. Both referees agree that your review is interesting and generally well-written. Nonetheless, both referees have provided several suggestions that are required to improve your review prior to acceptance. We look forward to receiving your revised manuscript.

REFeree COMMENTS

Referee #1:

General Comments

This is an interesting and timely review. Although the work is in general sound, I have some constructively critical points below that I hope will be of benefit to the authors in revising their work.

Specific Comments:

Line 147-148: Although MAFbx has been proposed to control protein synthesis by virtue of its binding to MyoD and eIF3-f, the authors are encouraged to read a nice review by Bodine and Baehr which challenges this interpretation based upon results from studies involving MAFbx knockout (Am J Physiol Endocrinol Metab. 307[6]: E469-484, 2014).

Lines 241-244: text relating to the impact of mitochondrial coupling. The authors state that mitochondrial uncoupling would increase mitochondrial ROS generation but depending upon the circumstances, uncoupled mitochondria may emit less ROS (for example, see PMID: 29859845). This should be clarified in the text.

Line 351-356: given that different exercise stimuli (grossly: resistance versus endurance) can have different impacts on skeletal muscle, it is important to specify the nature of the exercise stimuli in this statement. For example, although all types of exercise are considered to have some anti-inflammatory/antioxidant properties, not all are expected to yield a "potent hypertrophic stimulus". Please revise accordingly.

Line 362: "overreaching" - it is not clear what you mean here (over-training?).

Line 371-373: I am not sure that I agree that stimulating hypertrophy is the most intuitive benefit to be gained from exercise in the context of cancer cachexia. I would suggest that you mean antagonizing the atrophy signaling induced with cachexia (both atrophy signaling and suppression of protein synthesis). In other words, the goal of exercise in cancer cachexia is to attenuate the atrophy rather than generate hypertrophy (failure to get hypertrophy would not mean it is not effective/beneficial, right?).

Lines 384-386: I think this deserves a bit more consideration. For example, because eccentric muscle contractions cause more muscle damage, is it possible that this might push the muscle over the edge in the context of cachexia? Also, there appears to be multiple concepts related to eccentric muscle contractions here that should be better developed. First, there is the eccentric phase of any muscle contraction using 'free weight' as is relevant to a resistance or strength training stimulus. Second, there are special-built devices to generate eccentric muscle contractions such as braked eccentric bicycles that are used in the context of more of an endurance stimulus where reducing the cardiovascular demand is important (because as the authors point out, eccentric contractions are less energy demanding). Please expand.

Lines 440-443: what "recommendation" are you referring to here? Please specify.

Lines 447-450: the Wang et al. study used a genetic model of PGC-1a over-expression not a pharmacological approach. Please reconsider the interpretation in light of the point trying to be made in this sentence and the one that follows concerning conflicting work from Sandri's lab.

Line 460 (section on Insulin Resistance): the authors might also consider that exercise training would increase capillary angiogenesis and that may also contribute to an increased insulin sensitivity in the trained state (greater surface area for diffusion of insulin and glucose out of circulation into muscle fibers).

Line 497: immunodeficient mice are not needed for C26 or LLC models (e.g., see Geppert et al. Cancers 14: 90, 2022). Please confirm your source information and revise the text accordingly.

Line 494 (section on cachexia models): the challenges associated with mouse models of cancer cachexia and translating to humans is ongoing and unresolved. For starters, given the higher prevalence of cachexia with pancreatic cancer than other cancers (see Baracos et al. 2018), it does not seem unreasonable to think that different types of cancers might have different impacts on muscle because, for example, the cytokine profile released from the tumors may be different in important (but thus far unresolved) ways that influence the nature of the muscle impact. As such, I am not sure I would put as much stock in the conclusions from the Talbert et al. 2019 paper comparing different cancer models in terms of mirroring the cachexia occurring in humans with pancreatic cancer. This is an evolving area and I think it is important to expand on

what is known (a little) versus what remains unknown (a lot).

Referee #2:

Murphy and colleagues present a review article entitled "Impact of cancer cachexia on respiratory muscle function and the therapeutic potential of exercise". This manuscript includes an overview of mechanisms of muscle wasting, a systematic review of what is known about respiratory muscles and function in cancer cachexia, and a more conjectural discussion of how exercise might protect against muscle wasting in cancer patients. The manuscript is well-written, clear, and includes attractive figures. The authors correctly point out that little evidence exists to support respiratory muscle dysfunction in cancer patients or the more practical aspects of exercise interventions in cancer patients. My comments are generally minor in nature.

Major concern

While the authors thoughtfully discuss several aspects of preclinical models, it may be also beneficial to readers to note that the ability of exercise to reduce tumor burden in many of the traditional cancer cachexia models, for example:

<https://doi.org/10.1002/j.2617-1619.2018.tb00008.x>

While certainly not always true, disrupting or slowing tumor growth is often a missed mechanism for "preventing" cachexia - there are strong correlations between tumor burden and muscle mass, especially in very short-term animal models, and studies looking for benefits of exercise in pre-clinical models need to ensure that tumor burden is closely monitored before concluding that their interventions were effective.

Minor Concerns

A small quibble with the graphical abstract, in that it represents much smaller fibers in a muscle of the same size. While I appreciate that it's difficult to display visually, to me this implies a more dystrophic like phenotype of filling the space in muscle, and to my knowledge there is limited evidence for this being the case in cancer. Instead, muscles simply seem to take up less area.

While fair to say that long discussions of either of these points is beyond the scope of the review, comments on two minor points may benefit the reader: 1. most of what we know about exercise in people with cancer is actually in cancer survivors of either tumors with very high survival rates and/or cancer patients who have concluded cancer treatment - we have little knowledge of exercise in people who have cancer that is likely incurable, and these are the cancers most associated with cachexia, and 2. The difficulty of implementing an exercise intervention in patients undergoing active cancer treatment, whether real or perceived, has been a major barrier to investing in studying exercise interventions, either in animal models or in people. A number of feasibility trials are ongoing, but without data, we remain unsure if structured exercise programs are likely to be taken up by the majority of those likely to benefit.

In some places, it can be difficult to determine whether the authors are referencing a study conducted in humans or animal models. Similarly, if an exercise/exercise-like study was conducted in cancer patients or animals with cancer or instead is being used to support the likely benefits of exercise is sometimes unclear. While minor, a careful editing to ensure that when possible, key aspects of the referenced study are clear for individuals who are not experts in the field may benefit readers.

REQUIRED ITEMS:

- Author profile(s) must be uploaded via the submission form. Authors should submit a short biography (no more than 100 words for one author or 150 words in total for two authors) and a portrait photograph of the two leading authors on the paper. These should be uploaded, clearly labelled, with the manuscript submission. Any standard image format for the photograph is acceptable, but the resolution should be at least 300 dpi and preferably more. A group photograph of all authors is also acceptable, providing the biography for the whole group does not exceed 150 words.

END OF COMMENTS

Confidential Review

18-Jul-2022

Authors' Responses to Reviewing Editor and Referees

EDITOR COMMENTS

Reviewing Editor:

Thank you for submitting this topical review to the Journal of Physiology. Your report has been reviewed by two experts in the field. Both referees agree that your review is interesting and generally well-written. Nonetheless, both referees have provided several suggestions that are required to improve your review prior to acceptance. We look forward to receiving your revised manuscript.

RESPONSE: We are grateful to the Editors for their interest in the topic and our submitted article. We have addressed the referees' comments and have revised the original text, which has improved the manuscript. Thank you.

REFEREE COMMENTS

Referee #1:

General Comments

This is an interesting and timely review. Although the work is in general sound, I have some constructively critical points below that I hope will be of benefit to the authors in revising their work.

RESPONSE: We are grateful to the referee for their positive appraisal of our manuscript and for very helpful comments that have encouraged a revised manuscript adding considerable value to the work. Thank you.

Comment – Line 147-148: Although MAFbx has been proposed to control protein synthesis by virtue of its binding to MyoD and eIF3-f, the authors are encouraged to read a nice review by Bodine and Baehr which challenges this interpretation based upon results from studies involving MAFbx knockout (Am J Physiol Endocrinol Metab. 307[6]: E469-484, 2014).

RESPONSE: The recommended review article provided a detailed summary of the role of MuRF1 and MAFbx in skeletal muscle atrophy. Upon consideration of this information, it is evident that our initial statement (describing the control of protein synthesis via MAFbx) was not wholly accurate and was potentially misleading due to the erroneous certainty with which our statements were declared. The review article authored by Bodine and Baehr provided very valuable information. We have revised this section of the manuscript and we are very grateful to you for raising this point.

Comment – Lines 241-244: text relating to the impact of mitochondrial coupling. The authors state that mitochondrial uncoupling would increase mitochondrial ROS generation but depending upon

the circumstances, uncoupled mitochondria may emit less ROS (for example, see PMID: 29859845). This should be clarified in the text.

RESPONSE: In accordance with the above comment, we further explored the literature surrounding mitochondrial uncoupling and the generation of ROS and resultant implications for metabolic energy expenditure. We thank the reviewer for their suggestion and for highlighting the recommended article as it facilitated appropriate expansion of this subheading. Our initial statement in relation to mitochondrial uncoupling failed to reflect the complex implications of this process in relation to the generation of ROS. Furthermore, the potential implications of alterations in this process for muscle atrophy was not adequately illustrated to the reader. Therefore, additional information is now included, with the intention of succinctly summarising the mechanism and potential impact of mitochondrial uncoupling in cachexia.

Comment - Line 351-356: given that different exercise stimuli (grossly: resistance versus endurance) can have different impacts on skeletal muscle, it is important to specify the nature of the exercise stimuli in this statement. For example, although all types of exercise are considered to have some anti-inflammatory/antioxidant properties, not all are expected to yield a "potent hypertrophic stimulus". Please revise accordingly.

RESPONSE: The simplicity of the original statement is acknowledged. This section, which introduces the potential benefits of exercise, has been expanded to add clarity in relation to the differential effects of exercise dependent upon exercise type, the discussion of which is continued throughout the subheading.

Comment – Line 362: "overreaching" - it is not clear what you mean here (over-training?).

RESPONSE: The statement in question has been revised to include an appropriate definition of overreaching and to contextualise its relevance in this section of the manuscript.

Comment - Line 371-373: I am not sure that I agree that stimulating hypertrophy is the most intuitive benefit to be gained from exercise in the context of cancer cachexia. I would suggest that you mean antagonizing the atrophy signaling induced with cachexia (both atrophy signaling and suppression of protein synthesis). In other words, the goal of exercise in cancer cachexia is to attenuate the atrophy rather than generate hypertrophy (failure to get hypertrophy would not mean it is not effective/beneficial, right?).

RESPONSE: We fully agree with the above comment. Our description in the original text was misleading as the goal of exercise is indeed to attenuate atrophy. The manuscript has been revised accordingly. Thank you.

Comment - Lines 384-386: I think this deserves a bit more consideration. For example, because eccentric muscle contractions cause more muscle damage, is it possible that this might push the muscle over the edge in the context of cachexia? Also, there appears to be multiple concepts related to eccentric muscle contractions here that should be better developed. First, there is the eccentric

phase of any muscle contraction using 'free weight' as is relevant to a resistance or strength training stimulus. Second, there are special-built devices to generate eccentric muscle contractions such as braked eccentric bicycles that are used in the context of more of an endurance stimulus where reducing the cardiovascular demand is important (because as the authors point out, eccentric contractions are less energy demanding). Please expand.

RESPONSE: We appreciate the suggestion of the reviewer. Indeed, the topic of eccentric exercise interventions in clinical populations is vast and of particular interest in the context of pathologies wherein limiting energy expenditure is a priority. We believe our further exploration of this topic, encouraged by the referee, has added considerable value to the manuscript. We expand on the statements of the original manuscript, briefly introducing how eccentric exercise may provide greater benefit compared with concentric or isometric forms of training. Following this, the concept of moderate load eccentric training and Resistance Exercise via Negative Eccentric Work (RENEW) is discussed. Finally, the stimulus of exercise-induced muscle damage and the potential negative impact of the increase in this stimulus, as seen in eccentric contractions, is considered.

Comment - Lines 440-443: what "recommendation" are you referring to here? Please specify.

RESPONSE: Noted and clarified.

Comment - Lines 447-450: the Wang et al. study used a genetic model of PGC-1 α over-expression not a pharmacological approach. Please reconsider the interpretation in light of the point trying to be made in this sentence and the one that follows concerning conflicting work from Sandri's lab.

Answer – Noted and amended. Thank you.

Comment - Line 460 (section on Insulin Resistance): the authors might also consider that exercise training would increase capillary angiogenesis and that may also contribute to an increased insulin sensitivity in the trained state (greater surface area for diffusion of insulin and glucose out of circulation into muscle fibers).

RESPONSE: Thank you for raising this point. This is an interesting adaptation which we had not explored in the original manuscript. This subheading has been expanded to include information relevant to this important adaptation.

Comment - Line 497: immunodeficient mice are not needed for C26 or LLC models (e.g., see Geppert et al. Cancers 14: 90, 2022). Please confirm your source information and revise the text accordingly.

RESPONSE: Our reference to a need for immunodeficient animals in the use of subcutaneous models of cancer cachexia was misplaced. Such animals are typically, but not always, used in the induction of orthotopic models to reduce the probability of rejection. Thank you for pointing out this error, which has been corrected.

Comment - Line 494 (section on cachexia models): the challenges associated with mouse models of cancer cachexia and translating to humans is ongoing and unresolved. For starters, given the higher prevalence of cachexia with pancreatic cancer than other cancers (see Baracos et al. 2018), it does not seem unreasonable to think that different types of cancers might have different impacts on muscle because, for example, the cytokine profile released from the tumors may be different in important (but thus far unresolved) ways that influence the nature of the muscle impact. As such, I am not sure I would put as much stock in the conclusions from the Talbert et al. 2019 paper comparing different cancer models in terms of mirroring the cachexia occurring in humans with pancreatic cancer. This is an evolving area and I think it is important to expand on what is known (a little) versus what remains unknown (a lot).

RESPONSE: On consideration of the above comment, we acknowledge that the Preclinical Models of Cancer Cachexia subheading was framed poorly regarding our interpretation of the literature. Our intended message was not presented optimally. As noted by the referee, the original composition did not appropriately acknowledge the complexity of modelling cancer cachexia. It largely focussed on the published critiques of the subcutaneous models without adequately addressing our consideration of these critiques in the context of studying the impact of exercise in cancer cachexia.

In response, we have expanded the subheading to include additional information with the intention of affording the reader a more comprehensive appreciation of the diversity of models available and the challenges involved in utilising these models, in addition to providing the reader with a more coherent viewpoint of the argument in favour of the KPP model presented by Talbert and colleagues.

We thank the referee for this critique as addressing it has resulted in a much-improved subsection of the manuscript.

Referee #2

Murphy and colleagues present a review article entitled "Impact of cancer cachexia on respiratory muscle function and the therapeutic potential of exercise". This manuscript includes an overview of mechanisms of muscle wasting, a systematic review of what is known about respiratory muscles and function in cancer cachexia, and a more conjectural discussion of how exercise might protect against muscle wasting in cancer patients. The manuscript is well-written, clear, and includes attractive figures. The authors correctly point out that little evidence exists to support respiratory muscle dysfunction in cancer patients or the more practical aspects of exercise interventions in cancer patients. My comments are generally minor in nature.

RESPONSE: We are grateful to the referee for the positive appraisal of our manuscript. Specific comments are addressed below, and revisions made to the manuscript, based upon the constructive critique, have improved the article for which we are very grateful. Thank you.

Comment – “While the authors thoughtfully discuss several aspects of preclinical models, it may be also beneficial to readers to note that the ability of exercise to reduce tumor burden in many of the traditional cancer cachexia models. While certainly not always true, disrupting or slowing tumor growth is often a missed mechanism for "preventing" cachexia - there are strong correlations between tumor burden and muscle mass, especially in very short-term animal models, and studies looking for benefits of exercise in pre-clinical models need to ensure that tumor burden is closely monitored before concluding that their interventions were effective.”

RESPONSE: Thank you for raising this important point. Discussion of the potential considerable impact of exercise on tumour burden has been included as the first potential benefit of exercise explored under the Benefits of Exercise subheading. It is discussed as an overarching mechanism to maintain the logical flow of the manuscript, whereby the specific influence of mechanisms such as inflammation and oxidative stress are introduced in the Mechanisms of Cachexia subheading before being reconsidered in relation to the specific adaptations of exercise.

Comment – “A small quibble with the graphical abstract, in that it represents much smaller fibers in a muscle of the same size. While I appreciate that it's difficult to display visually, to me this implies a more dystrophic like phenotype of filling the space in muscle, and to my knowledge there is limited evidence for this being the case in cancer. Instead, muscles simply seem to take up less area.”

RESPONSE: Thank you for pointing this out. An appropriate revision has been made to the graphical abstract which better illustrates the reduction in overall muscle size in cancer cachectic patients.

Comment – “While fair to say that long discussions of either of these points is beyond the scope of the review, comments on two minor points may benefit the reader: 1. most of what we know about exercise in people with cancer is actually in cancer survivors of either tumors with very high survival rates and/or cancer patients who have concluded cancer treatment - we have little knowledge of exercise in people who have cancer that is likely incurable, and these are the cancers most

associated with cachexia. 2. The difficulty of implementing an exercise intervention in patients undergoing active cancer treatment, whether real or perceived, has been a major barrier to investing in studying exercise interventions, either in animal models or in people. A number of feasibility trials are ongoing, but without data, we remain unsure if structured exercise programs are likely to be taken up by the majority of those likely to benefit.”

RESPONSE: Although we had originally discussed the scarcity of human exercise research in cancer cachexia, we did not touch on the points highlighted here. Brief further discussion in the relevant introductory paragraph is now added, which further illustrates to the reader the difficulties involved in conducting exercise research in this clinical population. The growing support for exercise as therapy in cancer populations is noted. Complications involved in conducting such studies and the impact on assessment of end stage cancer patients is presented. Additionally, the inability of these results to inform the feasibility of cancer cachexia study due to the further potential contraindications of muscle wasting is acknowledged. Thank you for encouraging these revisions.

Comment – “In some places, it can be difficult to determine whether the authors are referencing a study conducted in humans or animal models. Similarly, if an exercise/exercise-like study was conducted in cancer patients or animals with cancer or instead is being used to support the likely benefits of exercise is sometimes unclear. While minor, a careful editing to ensure that when possible, key aspects of the referenced study are clear for individuals who are not experts in the field may benefit readers.”

RESPONSE: Thank you for pointing this out. We have revised the text to improve clarity for readers.

Dear Ken,

Re: JP-TR-2022-283569R1 "Impact of cancer cachexia on respiratory muscle function and the therapeutic potential of exercise" by Ken D O'Halloran, John J Mackrill, and Ben Murphy

I am pleased to tell you that your Topical Review article has been accepted for publication in The Journal of Physiology, subject to any modifications to the text that may be required by the Journal Office to conform to House rules.

NEW POLICY: In order to improve the transparency of its peer review process The Journal of Physiology publishes online as supporting information the peer review history of all articles accepted for publication. Readers will have access to decision letters, including all Editors' comments and referee reports, for each version of the manuscript and any author responses to peer review comments. Referees can decide whether or not they wish to be named on the peer review history document.

The last Word version of the paper submitted will be used by the Production Editors to prepare your proof. When this is ready you will receive an email containing a link to Wiley's Online Proofing System. The proof should be checked and corrected as quickly as possible.

All queries at proof stage should be sent to tjp@wiley.com

The accepted version of the manuscript will be published online, prior to copy editing in the Accepted Articles section.

Are you on Twitter? Once your paper is online, why not share your achievement with your followers. Please tag The Journal (@jphysiol) in any tweets and we will share your accepted paper with our 22,000+ followers!

Best wishes

Laura

Professor Laura Bennet
Senior Editor
The Journal of Physiology
<https://jp.msubmit.net>
<http://jp.physoc.org>
The Physiological Society
Hodgkin Huxley House
30 Farringdon Lane
London, EC1R 3AW
UK
<http://www.physoc.org>
<http://journals.physoc.org>

*** IMPORTANT NOTICE ABOUT OPEN ACCESS ***

To assist authors whose funding agencies mandate public access to published research findings sooner than 12 months after publication The Journal of Physiology allows authors to pay an open access (OA) fee to have their papers made freely available immediately on publication.

You will receive an email from Wiley with details on how to register or log-in to Wiley Authors Services where you will be able to place an OnlineOpen order.

You can check if your funder or institution has a Wiley Open Access Account here <https://authorservices.wiley.com/author-resources/Journal-Authors/licensing-and-open-access/open-access/author-compliance-tool.html>

Your article will be made Open Access upon publication, or as soon as payment is received.

If you wish to put your paper on an OA website such as PMC or UKPMC or your institutional repository within 12 months of publication you must pay the open access fee, which covers the cost of publication.

OnlineOpen articles are deposited in PubMed Central (PMC) and PMC mirror sites. Authors of OnlineOpen articles are permitted to post the final, published PDF of their article on a website, institutional repository, or other free public server, immediately on publication.

Note to NIH-funded authors: The Journal of Physiology is published on PMC 12 months after publication, NIH-funded authors DO NOT NEED to pay to publish and DO NOT NEED to post their accepted papers on PMC.

EDITOR COMMENTS

Reviewing Editor:

Thank you for your outstanding contribution to the Journal of Physiology.

Senior Editor:

Thank you for this very interesting and timely review.

REFEREE COMMENTS

Referee #1:

Thank you for your thorough revisions in response to my previous comments.

Referee #2:

Thanks to the authors for their thoughtful consideration of our criticisms and revisions to their manuscript. All of my concerns have been adequately addressed.

1st Confidential Review

29-Aug-2022